# Reduced *Drosophila suzukii* Infestation in Berries Using Deterrent Compounds and Laminate Polymer Flakes

**DOI:** 10.3390/insects8040117

**Published:** 2017-10-31

**Authors:** Justin M. Renkema, Rosemarije Buitenhuis, Rebecca H. Hallett

**Affiliations:** 1 School of Environmental Sciences, University of Guelph, 50 Stone Rd. E., Guelph, ON N1G 2W1, Canada; rhallett@uoguelph.ca; 2 Vineland Research and Innovation Centre, 4890 Victoria Ave. N., Box 4000, Vineland Station, ON L0R 2E0, Canada; rose.buitenhuis@vinelandresearch.com

**Keywords:** spotted wing drosophila, repellents, essential oils, thymol, citronellol, peppermint, strawberry

## Abstract

*Drosophila suzukii* (Matsumura) is a recent invasive pest of soft fruits in North and South America and Europe. Control relies on frequent applications of synthetic insecticides. Additional tactics are needed for development of an effective integrated pest management program. Study objectives were to evaluate the repellency and oviposition deterrent capability of compounds in plant essential oils and the effect of select compounds on infestation rates in strawberries, using laminate polymer flakes as a carrier. Of 14 compounds from 5 essential oils, thymol was the most repellent to adult *D. suzukii* males and females for up to 24 h in the laboratory. Citronellol, geraniol and menthol were moderately repellent. In a choice assay, thymol on cotton wicks adjacent to ripe raspberries reduced female fly landings and larval infestation levels. In a no-choice assay, thymol reduced female fly landings by 60%, larval infestation by 50% and increased fly mortality compared to controls. Neither citronellol alone nor a blend of four repellent compounds was as effective as thymol alone at reducing fly landing, larval infestation, or increasing fly mortality. In a choice assay using polymer flakes, larval infestation was greater in raspberries near untreated flakes than in raspberries near flakes treated with thymol or peppermint oil. In the field, thymol and peppermint flakes reduced larval infestation levels by 25% in strawberries at 4, but not 7, days after application, compared to untreated flakes. With future improvements in application strategies, deterrent compounds may have a role in improving the management of *D. suzukii*.

## 1. Introduction

*Drosophila suzukii* (Matsumura) (Diptera: Drosophilidae), spotted-wing drosophila, has recently become a common and serious pest of temperate fruit crops in many regions of the world [1]. Native to Asia, *D. suzukii* was first detected in California and Spain in 2008 and has since spread across North America, most of Europe and areas of South America [2,3,4]. Multiple, overlapping generations are the result of rapid population growth under optimal temperatures during summer months in most of the affected regions [5,6]. Crop damage is caused when adult female flies, using a serrated ovipositor, lay eggs in ripe and partially ripe fruit before harvest. Developing larvae cause soft, unmarketable fruit, leading to increased risk of secondary rot infections [7]. Economic losses of 80% yield or 20–37% revenue have been reported [8,9].

Integrated pest management to reduce *D. suzukii* populations and prevent economic damage relies on the combined use of several strategies. Chemical insecticides provide rapid-knockdown of adult flies, and adjuvants (e.g., sucrose), as well as the optimal timing of applications, may improve the efficacy, and reduce the number, of sprays per season [10,11]. Field sanitation—the removal of dropped fruit and overripe hanging fruit—and managing field-margin, non-crop host plants may reduce *D. suzukii* populations [12]. In open-field or semi-protected berry plantings, exclusion netting prevents flies from accessing ripe fruit [13,14]. Natural enemies can provide some reduction of *D. suzukii* populations and development of sterile insect techniques through genetic modification is underway, but programs have not yet been developed for operational use by growers [15,16,17,18,19]. To reduce reliance on synthetic insecticides—which may lead to resistance development or suppression of natural enemy communities—and build an effective integrated pest management strategy, new tools against *D. suzukii* are needed.

Naturally-occurring plant compounds provide insects with critical olfactory information about their environments and are used by insects to mediate feeding and oviposition behavior. *Drosophila suzukii* attraction to ripe fruit is relatively unique among *Drosophila* spp., as it is responsive to volatiles associated with fruit-ripening and exhibits behavioral preference for green-leaf volatiles, particularly β-cyclocitral [20]. On the other hand, certain volatile phytochemicals can elicit host-avoidance behavior in insects. Plant essential oils have been effective at repelling herbivorous pests from crop hosts, including codling moth larvae (*Cydia pomonella* (L.)), red bud borer midges (*Resseliella oculiperda* (Rüsaamen)) and western flower thrips (*Frankliniella occidentalis* (Pergrande)) [21,22,23]. Essential oils that interfere with *D. suzukii* host-finding ability or deter them from oviposition sites, and thus reduce crop damage, may be particularly useful in conjunction with other tactics (e.g., the ‘push-pull’ system; [24]), especially in organic small fruit production, where current management strategies are limited.

In recent studies, a handful of repellent or deterrent compounds have been tested against *D. suzukii*. In the laboratory, geosmin and 1-octen-3-ol were repellent to female flies up to a 10-fold dilution [25]. Butyl-anthranilate, a compound found in some ripening fruits, reduced oviposition by nearly 50% when applied at 2.5% to blueberries in the laboratory [26]. Of 12 essential oils tested, thyme and peppermint oils resulted in the highest levels of repellency in choice assays [27], and lavender floral oil was found to be an effective fumigant and contact repellent [28]. Potassium metabisulfite, an antimicrobial in food processing, was not repellent but was slightly toxic at high concentrations [27]. Citronellal, a compound in some plant essential oils, was not repellent to *D. suzukii*, while other major compounds, such as linalool and 1,8-cineole, were [26,28]. Further identification of repellent compounds from essential oils may improve the practical and economic feasibility of using repellents for management of *D. suzukii*, and blends of the most repellent compounds may improve efficacy.

A major challenge to developing a deterrent technique for *D. suzukii* is finding an appropriate and effective field dispersal method, as spraying essential oils on plants and fruit—even at 1–2%—can cause phytotoxicity and unwanted residues [29]. Research into non-spray application methods showed that 1-octen-3-ol provided nearly a 50% reduction in eggs in raspberries when it was in small dispensers close to ripening fruit clusters, and a 30–50% reduction when formulated with a specialized pheromone and lure application technology (SPLAT) in hangtags placed at a density of 10 per m^2^ [25,30]. Laminate flake technology has been used to disperse insect pheromones in agriculture for mating disruption (e.g., [31]) and may be a practical carrier for *D. suzukii* repellents. With flakes that can be quickly dispersed over a large area, repellent volatiles will be emitted from many small point-sources. Flakes may be particularly useful in low-growing berry crops, such as strawberries, as flakes falling on the ground or foliage will remain proximal to ripening fruit.

The first objective of the current study was to evaluate the repellency of major compounds present in plant essential oils that were previously shown to be repellent to *D. suzukii* flies [27]. Compounds that were repellent were then tested individually or in a blend for their ability to deter *D. suzukii* oviposition in berries. The second objective was to evaluate the effect of most deterrent compounds or blends formulated in laminate polymer flakes (Disrupt Bio-flakes^®^, Hercon Environmental) against *D. suzukii* in the laboratory and a strawberry field.

## 2. Materials and Methods

### 2.1. Insect Colony

Laboratory assays were conducted with *D. suzukii* from a laboratory colony maintained since 2012 at the University of Guelph in ventilated Plexiglas^®^ cages (26 × 26 × 26 cm) on rearing media. Media was agar (45 g), cornmeal (125 g), white sugar (200 g), and nutritional yeast (70 g) with 1 M propionic acid (17.7 mL) and methyl paraben (3.3 g) dissolved in boiling water (4 L), cooled and poured into Petri dishes. Flies were given moist cotton as a water source and fresh media twice weekly. The colony was started with *D. suzukii* that emerged from infested raspberries and blackberries collected at a commercial farm near Halton Hills, ON, Canada (43°34′43″ N; 79°57′48″ W).

### 2.2. Repellency of Essential Oil Compounds Using Choice Bioassays

Major compounds (defined as >5% of total) in the essential oils of citronella (*Cymbopogon winterianus* Jowitt), eucalyptus (*Eucalyptus radiata* Spreng.), geranium (*Pelargonium asperium*/*P. graveolens* (L.) L’Her ex Ait, cv. Bourbon or Rosat), peppermint (*Mentha* × *piperita* L.) and thyme (*Thymus vulgaris* L.) were identified based on gas chromatography (GC) analyses provided by an essential oil supplier (Aliksir Inc., Grondines, QC, Canada) and/or in published literature (Table 1), and were purchased from Sigma-Aldrich at highest available purities (≥95 to 99%, except isomenthone at 90%).

Bioassays were set up following methods described by Renkema et al. [27]. Briefly, the full essential oil, each of the major compounds, and a blend of the major compounds in each oil were presented on cotton dental wicks held in glass vials to male and female *D. suzukii* flies in ventilated plastic containers (arenas). The vials were 18.7 cm apart and 7.3 cm from the ends of arenas. One wick contained organic, fresh-pressed, pure blueberry juice (Lakewood Organic Juices, Miami, FL, USA) plus the repellent compound dissolved in acetone and the other contained juice plus acetone. Full essential oils were applied at a rate of 10 mg/wick in 2 mL of acetone, each compound was applied at the percentage found in the full essential oil, and the blend at the sum of the percentages of all major compounds in the oil (see Table 1). For example, citronella oil was tested at 10 mg/wick, citronellal at 3.4 mg/wick, geraniol at 2.2 mg/wick, citronellol at 1.2 mg/wick and the 3-compound blend at 6.8 mg/wick. Assays were conducted separately for male and female flies, with 20 flies per arena, and after 2, 6, and 24 h, the location of flies in each arena, on treated versus untreated wicks, and numbers alive or dead were recorded.

### 2.3. Oviposition Deterrence of Repellent Compounds Using Choice and No-Choice Bioassays

To test whether compounds that repelled *D. suzukii* also deterred oviposition, choice and no-choice bioassays were conducted. Cotton dental wicks (3.5 cm length) were secured to the bottoms of 5 cm diameter Petri dishes with hot glue. Wicks were treated with compounds or compound blends in acetone (2 mL per wick) or acetone only (see Table 2 for amounts) and placed in a fumehood for one hour to allow acetone to volatilize. There were three treatments: a four-compound blend with amounts of each compound the same as in the repellency assay (Section 2.2); a thymol-dominated blend; and a citronellol-dominated blend. Dishes with treated wicks were secured to the bottom of the same arenas used in the previously described assay with a small piece of mounting putty (Lepage, Henkel Canada Corp., Mississauga, ON, Canada). For the choice assay, there were two Petri-dishes on opposite ends of the container (18 cm apart), and in no-choice assay there was one dish 7 cm from one end. Store-purchased red raspberries were dipped in 1% bleach solution for 15 s (to kill microorganisms), followed by water for 5 s and placed on paper towel to dry. Two raspberries were weighed to have consistent sizes among replicates and placed open end down in each dish, on either side of, but not in contact with, the cotton wick.

*Drosophila suzukii* flies that emerged over a three day period were placed into “mating chambers” [43] for 2 d at a ratio of 5:3 female:male. A chamber was a plastic centrifuge tube (500 mL) with a 2 cm diameter hole cut into one side and covered with fine mesh for ventilation. A 1 cm diameter hole was cut into the opposite side and filled with moist cotton to provide a water source. A piece of rearing diet (~3 g) was placed in the lid [44]. After 2 d, all flies from mating chambers were released into a single Plexiglas cage and five females aspirated into a plastic vial (70 mL). Vials were placed upright in the center of each arena, the vial lid removed, and arena lids quickly closed. Arenas were arranged in a complete randomized design, and the end of the arena with the treated wick (in choice assay) and the single wick (in no-choice assay) was alternated in adjacent arenas. The choice experiment was conducted twice, 23–24 July and 30–31 July 2014 to obtain 10–12 replicates per treatment.

The number of flies on treated or control wicks, those dead in the arena, and those alive or dead in the release vial were counted 6 and 24 h after flies were introduced. After 24 h, raspberries were removed from the Petri-dishes and held in plastic vials (100 mL) in the laboratory for 4 d to allow eggs to hatch and larvae to develop. A saltwater test [45] (1:16 *v*/*v* salt:water) was used to float and count larvae from gently crushed raspberries.

### 2.4. Repellency and Oviposition Deterrence of Compounds in Biopolymer Flakes Using a Choice Bioassay

Bio-flakes^®^ were formulated by the manufacturer (Hercon Environmental) with ~10% *w*/*w* peppermint oil (Jedwards International Inc., Braintree, MA, USA) or thymol (Sigma-Aldrich Inc., St. Louis, MO, USA) or left unformulated (untreated). Peppermint oil was included because it performed well in our previous study [27] and Hercon Environmental was testing it for other applications. Release rates of peppermint oil and thymol were calculated by placing ~170 g of formulated Bio-flakes^®^ in a drying oven at 30 °C and recording the weight 0, 7, and 14 or 15 d later.

*Drosophila suzukii* flies were placed in mating chambers as described above for bioassays, except that male flies were removed 10 h prior to the start of the experiment and the lid with diet was replaced with a lid without diet. Diet was removed so that food-deprived flies would respond more quickly to food odors in olfactometers. Female flies were transferred from the mating chambers into vials immediately prior to release into experimental arenas.

Two I-tube olfactometers (clear, acrylic cylinders, 12 cm inside diameter × 120 cm length; 3 mm thickness; 2 tubes) were set-up in a window-less laboratory room side by side (36 cm apart) on tables covered with white paper. The olfactometers were supported 10 cm above the table on white Styrofoam holders, positioned 10 cm from each end of each I-tube. A white sheet was hung 20 cm behind each end of the I-tubes to minimize external visual stimuli. Two incandescent light bulbs, positioned evenly between the I-tubes, were hung 40 cm above and at each end of the I-tubes.

In the middle of each I-tube, two 3.5 cm diameter holes were made opposite each other. Flies were introduced through the top hole, and a mesh covered black rubber stopper with a 5 mm hole was fitted into the bottom hole. An AirAdmiral^®^ (Cole-Parmer Instrument Co., Model# P-79202-00; 10.5 LPM; Montreal, QC, Canada) air compressor was attached with 6.35 mm tubing to the hole in the stoppers to constantly pull air through both I-tubes simultaneously. A moist cotton ball was placed in the I-tube below the fly entry point to provide moisture for flies during the experiment. The ends of the I-tubes were closed with white mesh secured around the I-tube with rubber bands.

A 1.5 cm diameter × 4.5 cm long glass vial was secured upright to the middle of a 6 cm diameter glass Petri dish with a small piece of mounting putty. Bio-flakes^®^ (10 g) treated with thymol or peppermint oil or blank Bio-flakes^®^ were placed in the dish around the vial. A fresh, store-purchased, red raspberry was rinsed in 1% bleach followed by water and allowed to air dry before it was weighed and placed on the top, open end of the vial, above the flakes. For each replicate, the end of the tube and the end of the table with treated Bio-flakes^®^ (40 cm from center of tube) was randomly selected. The other side received blank Bio-flakes^®^. Petri-dishes were held to the bottom of the I-tube with two small pieces of mounting putty. Between replicates, the inside of the I-tube was cleaned with acetone on Kimwipes^TM^ (Kimberly-Clark Co.; Irving, TX, USA) and allowed to dry 

Replicates (8 for thymol and 9 for peppermint oil) were conducted during March and April 2015. Eight female flies were released into each I-tube from a 3.2 cm diameter × 7 cm long plastic vial (bottom replaced by mesh) that was fitted into the top hole in each I-tube. Flies were tapped from the vial into the I-tube. I-tubes were divided into zones, 0–10 cm, 10–25 cm, 25–40 cm, >40 cm, in each direction from the central entry hole. The location (i.e., zone or on raspberry) of each live fly was recorded 2, 6, 10, and 22 h after their introduction. Replicates with more than 1 dead fly after 22 h were not used for analysis. After 22 h, raspberries were removed and placed in 50 mL glass beakers with a piece of mesh secured with a rubber band over the beaker. Beakers were held in the laboratory for 5 d to allow larvae to develop. Larvae were extracted from raspberries and counted using a saltwater test.

### 2.5. Oviposition Deterrence of Compounds in Biopolymer Flakes in the Field

Bio-flakes^®^ formulated with 10% *w*/*w* peppermint oil, thymol or left unformulated (untreated) were applied to commercial day-neutral strawberries, (*Fragaria* × *ananassa* Duchesne ‘Albion’) near London, Ontario (43°2′25″ N, 81°7′59″ W). Strawberries were grown in raised beds covered by white plastic according to standard practices. Insecticides were applied by the grower throughout harvest (beginning mid-July) for control of *D. suzukii*, with a final application of Delegate^TM^ (spinetoram) (Dow AgroSciences Canada Inc., Calgary, AB, Canada) at 280 g/ha in mid-August (2 weeks before the experiment) in the 0.6 ha field used for this experiment.

Plots were 2 × 0.6 m and arranged in five blocks, with each block along one raised bed (2 rows of strawberries) and plots separated by a 2–3 m buffer. Block location in the field was randomly chosen, with at least 5 m between blocks. Weedy areas with poor plant growth were not used.

On the same day Bio-flakes^®^ were applied, all ripening, ripe and overripe strawberries and strawberries fallen in the aisles on either side of each block (plot + buffer area), as well as any weeds, were removed by hand. On 4 September, Bio-flakes^®^ (21.6 g/plot) were shaken out of a plastic container (120 mL), distributing them evenly over the strawberry foliage in each plot. The foliage was then lightly disturbed by running a hand through the leaves so that Bio-flakes^®^ fell below the canopy, nearer the developing strawberries.

All ripe strawberries were hand-picked from plots on 8 and 11 September 2014 and weighed. Strawberries were held in covered, ventilated plastic cups (500 mL) for 3–4 d at 23–25 °C before saltwater was used to extract and count larvae. A sample of living larvae (30/plot/harvest) were transferred from saltwater to rearing media in Petri dishes that were held at room temperature for 10 d. The number of emerged *D. suzukii* and other *Drosophila* spp. flies were counted.

### 2.6. Data Analysis

For choice bioassays, the numbers of *D. suzukii* on treated and untreated wicks and on or in raspberries near treated or untreated wicks or Bio-flakes were compared using goodness-of-fit G-tests. The pooled G-test statistic was used because results were consistent among replicates (heterogeneity G-test, *p* > 0.05) [46].

For no-choice bioassays, analysis of variance (ANOVA) was used to compare numbers of live or dead *D. suzukii* on or in raspberries or strawberries near wicks or Bio-flakes^®^ treated with different compounds or left untreated. In order to combine replicates from each of the two times the assay was conducted, larval infestation rates were standardized by dividing the number of larvae per each raspberry pair by the total larvae in all arenas on each assay date. In all cases, data was normally distributed, error variance was homogeneous and data transformations were not used. Means were separated using Tukey’s HSD test.

Release rates from Bio-flakes^®^ were modelled using non-linear exponential decay functions, and number of days to 50% active compound remaining were predicted. JMP software [47] was used for ANOVAs (α = 0.05) and non-linear modelling.

## 3. Results

### 3.1. Repellency of Essential Oil Compounds Using a Choice Bioassay

**Figure 1:**
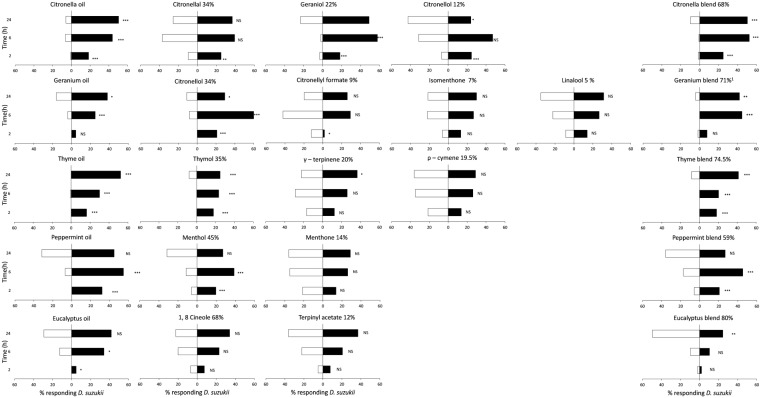
Percentage of male *Drosophila suzukii* on cotton wicks with blueberry (filled bars) or blueberry juice + repellent compound(s) (empty bars) at 2, 6 and 24 h after start of experiment. Oils applied at 10 mg/cotton wick, individual compounds at the proportion in which they occurred in the oil (i.e., citronella at 3.4 mg/wick), and blends at the sum of the proportions of individual compounds. Significant differences (G-test): *** (*p* < 0.001), ** (*p* < 0.01), * (*p* < 0.05), ‘NS’ no significant difference (*p* > 0.05).

**Figure 2:**
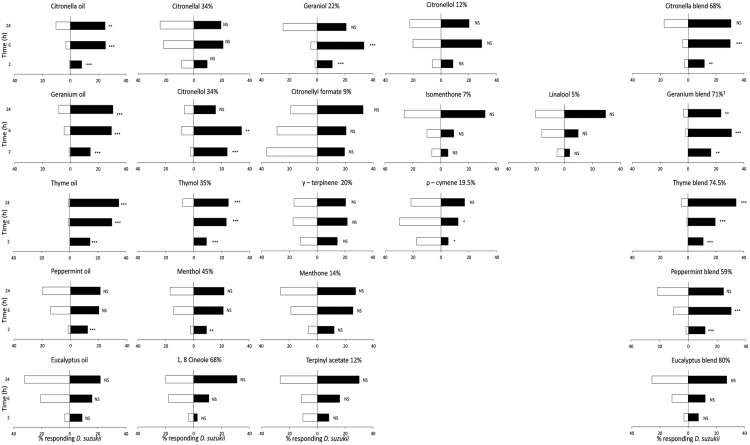
Percentage of female *Drosophila suzukii* on cotton wicks with blueberry (filled bars) or blueberry juice + repellent compound(s) (empty bars) at 2, 6 and 24 h after start of experiment. Oils applied at 10 mg/cotton wick, individual compounds at the proportion in which they occurred in the oil (i.e., citronella at 3.4 mg/wick), and blends at the sum of the proportions of individual compounds. Significant differences (G-test): *** (*p* < 0.001), ** (*p* < 0.01), * (*p* < 0.05), ‘NS’ no significant difference (*p* > 0.05).

Citronella oil, geranium oil and thyme oil were repellent to both male and female flies for at least 24 h (Figure 1 and Figure 2). However, thymol was the only individual chemical compound tested that showed a highly significant level of repellency to male and female *D. suzukii* for 24 h. Citronellol, the major compound in geranium oil (34%), and geraniol were significantly repellent to female flies at 6 h but lost repellency by 24 h. At 24 h, there was a modest level of repellency to citronellol and geraniol for male *D. suzukii*. Menthol was highly repellent to male *D. suzukii* at 6 h, but lost repellency at 24 h and was not repellent to females. All other individual compounds tested did not show repellency to *D. suzukii* males or females. Blends of major compounds from each oil resulted in similar levels of repellency to both males and females as achieved with the full oil, except for citronella oil and its blend against female *D. suzukii*. At 24 h, no repellency to females was recorded with the blend, whereas citronella oil was moderately repellent (Figure 2).

### 3.2. Oviposition Deterrence of Repellent Compounds Using Choice and No-Choice Bioassays

**Figure 3:**
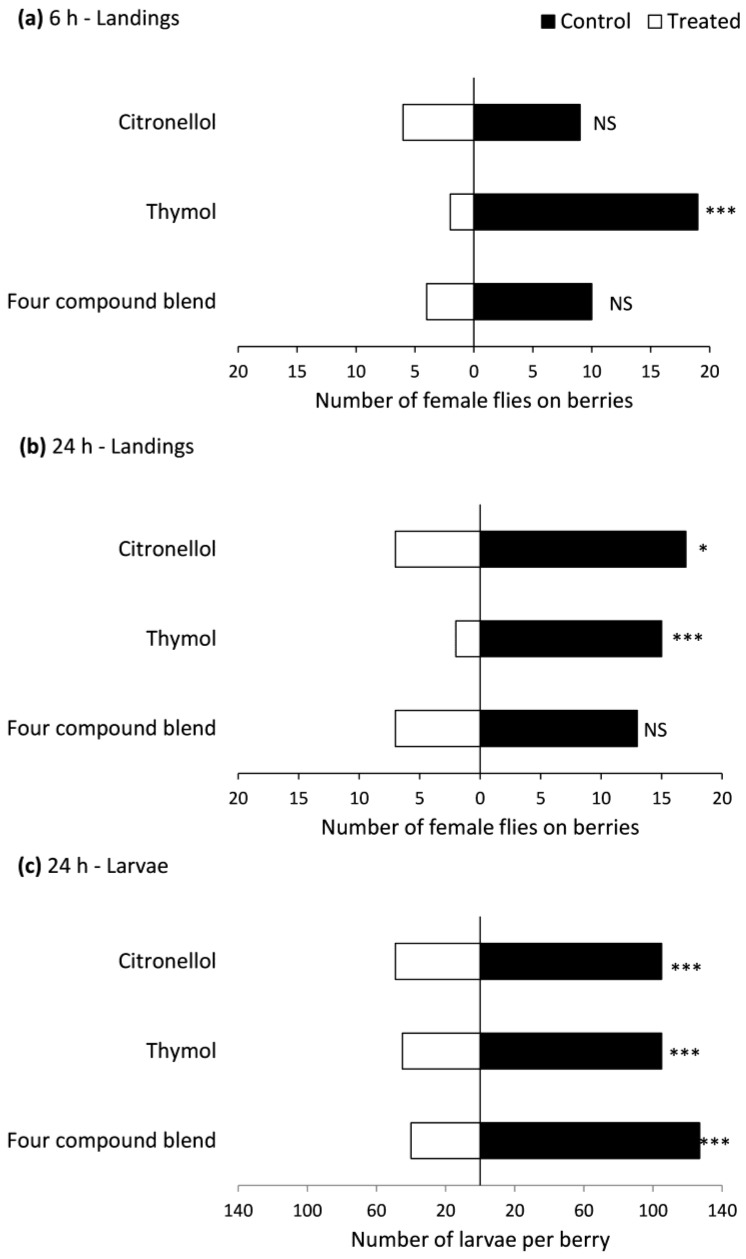
Number of female *Drosophila suzukii* flies landing at 6 h and 24 h after the start of the experiment on (**a**,**b**) and number of larvae recovered in (**c**) raspberries next to cotton wicks treated or not treated with repellent compounds. Significant differences (G-test): *** (*p* < 0.001), ** (*p* < 0.01), * (*p* < 0.05), ‘NS’ no significant difference (*p* > 0.05).

In the choice bioassay, fewer female flies landed on raspberries near thymol-treated than untreated wicks at 6 and 24 h after the start of the experiment, but treatment with a four-compound blend did not affect landings, and treatment with citronellol had only modest effects on landings (Figure 3a,b). Numbers of larvae recovered in raspberries near wicks treated with thymol, citronellol and a four-compound blend were significantly lower than in raspberries near untreated wicks, with 68–76% larvae found in the latter (Figure 3c). There were no differences among treatments in numbers of dead flies at 24 h: 1.3 ± 0.47, 1.4 ± 0.45, 1.2 ± 0.33 (±SEM) for citronellol, thymol and the four-compound blend, respectively (*F* = 0.1, df = 2, 27, *p* = 0.946).

**Figure 4:**
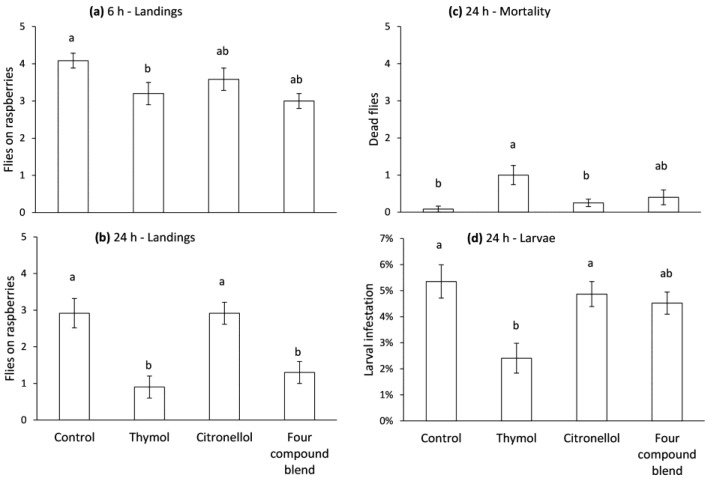
Number of female *Drosophila suzukii* flies landing on (**a**,**b**), number of dead flies on (**c**) and level of larval infestation in (**d**) raspberries next to cotton wicks either treated or not treated (control) with repellent compounds. Means with the same letter in each panel are not significantly different, Tukey’s HSD test (*p* > 0.05).

In the no-choice bioassay, fly landings were affected by treatment at 6 h (*F* = 3.0, df = 3, 40, *p* = 0.041) and 24 h (*F* = 10.0, df = 3, 40, *p* < 0.0001) after the start of the experiment. Fewer flies landed on raspberries near thymol-treated wicks than untreated wicks at 6 h, and fewer flies landed on raspberries near wicks treated with thymol and the four-compound blend than those near citronellol-treated or untreated wicks (Figure 4a,b). Mortality was also affected by treatment (*F* = 4.9, df = 3, 40, *p* = 0.005). There were more dead flies in arenas with thymol-treated wicks than those with untreated wicks (Figure 4c). Larval infestation was affected by treatment (*F* = 5.6, df = 3, 40, *p* = 0.003), with lower infestation in raspberries near thymol-treated wicks than those near untreated wicks (Figure 4d).

### 3.3. Repellency and Oviposition Deterrence of Compounds in Biopolymer Flakes Using a Choice Bioassay

**Figure 5:**
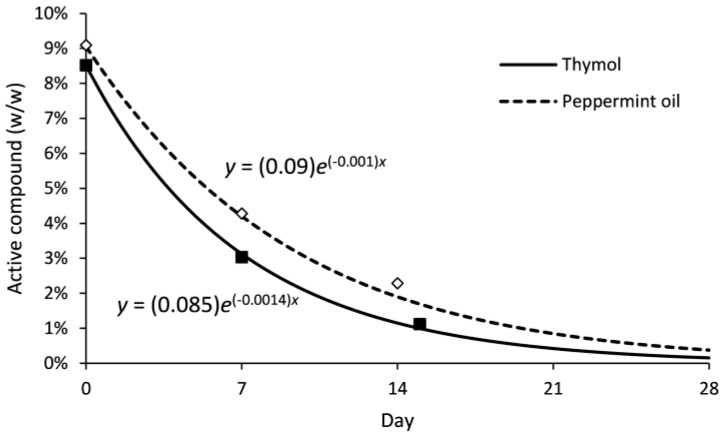
Release rate (%*w*/*w*) of thymol and peppermint oil formulated Bio-flakes^®^ at 30 °C. Non-linear regressions fitted to the data.

Similar release rates were recorded for thymol and peppermint oil in Bio-flakes^®^ (Figure 5). Exponential decay functions predicted a 50% loss of thymol after 4.8 d and of peppermint oil after 6.7 d.

**Figure 6:**
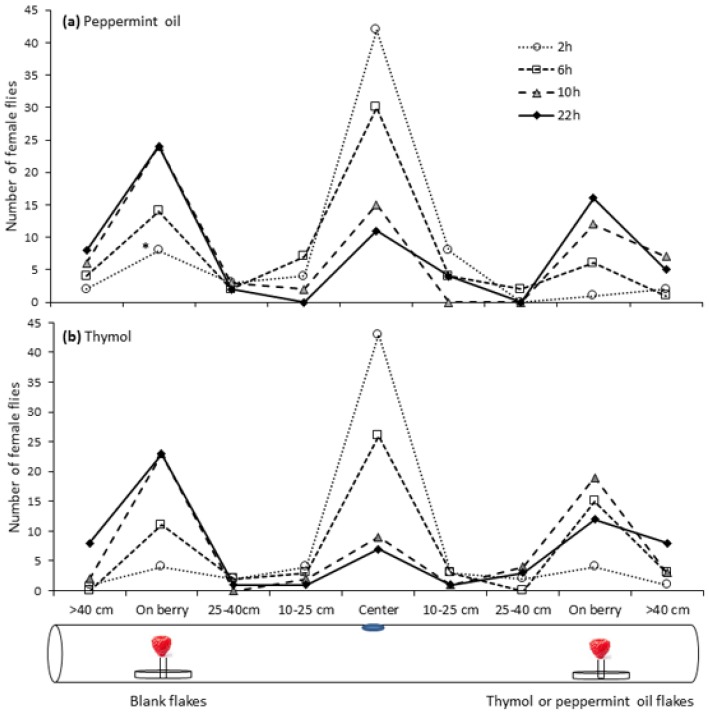
Location of female *Drosophila suzukii* flies in arenas at 2, 6, 10 and 22 h when Bio-flakes^®^ treated with either peppermint oil or thymol were compared to untreated Bio-flakes^®^ and placed in Petri-dishes below raspberries.

*Drosophila suzukii* female flies did not exhibit a strong preference when given a choice between raspberries placed above thymol- or peppermint oil-treated Bio-flakes^®^ and blank Bio-flakes^®^ (Figure 6). The numbers of flies on raspberries above blank Bio-flakes^®^ were numerically higher than numbers on raspberries above treated Bio-flakes^®^ at most observation times, but there was only a statistically different number of flies on blank compared to peppermint oil Bio-flakes^®^ at 2 h (*p* > 0.05 for all other observations for thymol and peppermint oil). Few flies were observed on the tube in the zones between the central entry hole and the raspberry or beyond the raspberry (Figure 6).

**Figure 7:**
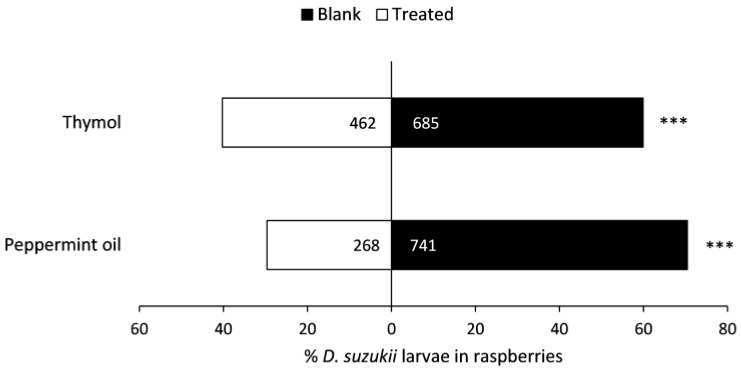
Numbers of *Drosophila suzukii* larvae recovered in raspberries near Bio-flakes^®^ treated or not treated (blank) with repellent compounds in a laboratory choice bioassay. Significant differences (G-test): *** (*p* < 0.001).

There were fewer larvae extracted from raspberries that had been held above thymol- or peppermint oil-treated flakes compared to those held above blank Bio-flakes^®^ (Figure 7). Since weights of paired raspberries in each tube and average raspberry weights among replicates were similar, conducting analyses with larvae per gram of raspberry did not affect results.

### 3.4. Oviposition Deterrence of Compounds in Biopolymer Flakes in the Field

**Figure 8:**
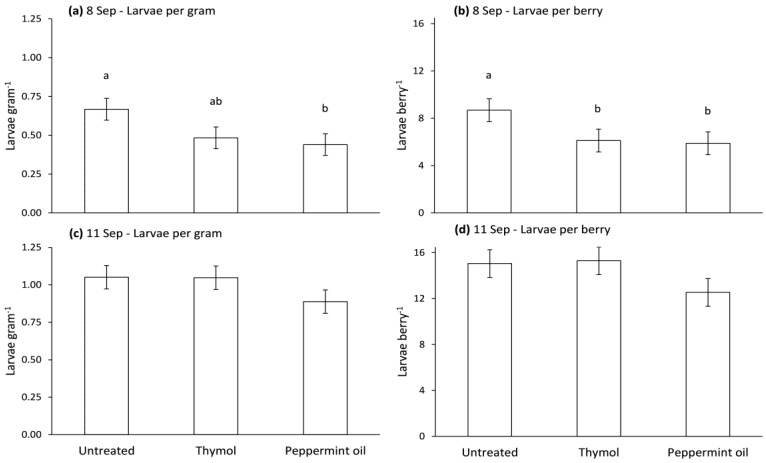
Mean (±SE) numbers of drosophilid larvae per gram of strawberry or per strawberry. Strawberries were picked 8 (**a**,**b**) or 11 (**c**,**d**) September 2014 from plots treated 4 September with untreated or thymol or peppermint treated (10% *w*/*w*) Bio-flakes^®^. Means with the same letter in each panel are not significantly different, Tukey’s HSD test (*p* > 0.05).

Numbers of drosophilid larvae per gram of strawberries and per strawberry were significantly affected by treatment on 8 September (*F* = 6.0, df = 2, 8, *p* = 0.0254; *F* = 6.9, df = 2, 8, *p* = 0.018), but not for those picked 11 September (*F* = 1.8, df = 2, 8, *p* = 0.2462; *F* = 2.4, df = 2, 8, *p* = 0.17). On 8 September, numbers of larvae per gram of strawberries were lower in strawberries from plots treated with Bio-flakes^®^ containing peppermint oil than in plots with blank Bio-flakes^®^, and numbers of larvae per strawberry were lower in strawberries from plots treated with Bio-flakes^®^ with peppermint oil or thymol than in plots with blank Bio-flakes^®^ (Figure 8).

From strawberries picked 8 September, flies emerged from 91% of larvae reared on media, and 100% of these flies were *D. suzukii*. On 11 September, flies emerged from 73% of larvae reared on media, and 99.7% of these flies (i.e., all but one) were *D. suzukii*.

## 4. Discussion

Using plant-derived essential oils or their constituent compounds to repel *D. suzukii* from or deter oviposition in fruit may reduce or delay in-field population build-up. Complete control of *D. suzukii*, even with frequent insecticide use, is challenging [48]; deterrents could be used to lengthen insecticide spray intervals or as part of a push-pull system to protect fruit crops. Deterrent compounds fit well in an integrated pest management and may be more compatible than insecticides with biological control options that are under development for *D. suzukii* [30,49]. While levels of reduction in *D. suzukii* infestation rates caused by repellent compounds in our experiments were modest and are not recommended as a stand-alone strategy, thymol and possibly peppermint oil warrant further testing as part of an integrated pest management strategy against *D. suzukii*. Methodological developments to overcome in-field deployment limitations of highly volatile compounds, such as those from essential oils, are needed.

Thymol (2-isopropyl-5-methylphenol) was identified as the major constituent in thyme oil responsible for repellent and deterrent activity. Other major compounds in thyme oil, γ-terpinene (20%) and ρ-cymene (20%), had no repellent activity. Thymol alone at the same amount as in the full oil and when blended with γ-terpinene and ρ-cymene resulted in nearly the same level of repellency as with the full oil for male and female *D. suzukii*. Results are consistent with our previous essential oil assays where thyme oil was one of the most repellent oils, tended to lower the number of flies responding (choosing either treated or untreated wicks), and exhibited a low-level of toxicity [27]. Thymol is highly toxic to other agricultural insect pests [50,51,52], and toxic neurological effects on *Drosophila melanogaster* Meigen have been described [53]. However, further understanding of its biochemical properties that cause non-lethal deterrence from a food source or testing concentrations that cause greater levels of toxicity may improve its use against *D. suzukii*. The source of, and extraction method used for, thyme oil may affect the presence and ratios of constituent compounds [54], therefore, the use of thymol, a single molecule that can be synthesized, represents an improvement in developing a consistent repellent. Thymol is used as an additive in the food industry for general antiseptic purposes, has demonstrated benefits in agriculture against bacteria, fungi, nematodes and other insects [55,56,57], and is exempt from requiring a pesticide chemical residue tolerance by the United States Environmental Protection Agency for certain applications (40 CFR Part 180, 6 June 2003). Thus, thymol is a good candidate molecule for development as a repellent or deterrent for *D. suzukii* in berry and fruit crops.

Even though neither peppermint oil nor its major compounds, menthol and menthone, provided good repellency in our initial wick choice assay, the level of deterrence and reduction in fruit infestation when it was formulated in Bio-flakes^®^ were equal to or slightly better than that achieved with thymol. Peppermint oil was highly repellent in our previous assays, likely attributable, at least in part, to a slower release rate than other oils [27]. Peppermint oil has proven, prolonged repellency to other pest flies (Diptera), and pennyroyal oil, *Mentha pulegium* L., is toxic to *Drosophila auraria* Peng [58,59,60]. Low response rates by female flies in our previous study indicate that the active space of peppermint oil may be greater than other oils, keeping flies further from oviposition sites [27]. While lower fly landing rates in our experiments generally resulted in lower larval counts.

Volatility plays an important role in determining the repellent activity of candidate compounds. Wallingford et al. [30] estimated a rate of at least 10 mg/h may be biologically relevant for *D. suzukii*; however, amounts of peppermint oil or thymol released fell short of this rate. Wallingford et al. [30] achieved 10 mg/h for the first 24 h with a 20% octenol formulated in a 2–3 g SPLAT hangtag. In the laboratory assay (22 h) with 10 g of Bio-flakes^®^, 3.83 and 4.71 mg were released per hour for peppermint oil and thymol, respectively. In the field, 198.7 and 244.1 mg of peppermint oil and thymol, respectively, were released over the entire plot (1.2 m^2^) in the first 24 h. Release rates for Bio-flakes^®^ were calculated at 30 °C and are probably reduced at lower temperatures or fluctuate with changing moisture levels in the field. Since the maximum amount of active compound in Bio-flakes^®^ is about 10% *w*/*w*, higher release amounts can be achieved by increasing the weight of Bio-flakes^®^ per unit area. In addition, the size of individual Bio-flakes^®^ may affect release rates and amounts and should be optimized in the future. 

A goal of this study was to identify repellent compounds from essential oils that could be combined to create a blend with greater deterrence than any oil or compound alone. However, a blend of thymol, citronellol, menthol, and geraniol did not improve deterrence over using thymol alone. Failure of this blend to outperform thymol may be due to the fact that all three other compounds are major constituents of less volatile essential oils [27]. Certain minor compounds in essential oils may act as synergists with major compounds to improve toxicity or feeding deterrence, such as trans-anethole with thymol against tobacco cutworms (*Spodoptera litura* F.) [50]. Further work should be conducted to determine an optimal two- or three-component blend based on the individual compounds that have been identified as good *D. suzukii* repellents by different research groups to date [25,26,27,28,30]. Mixtures of highly volatile and repellent compounds with more persistent repellent properties may improve longer-term efficacy in the field.

The success of deterrents against *D. suzukii* and their inclusion in integrated pest management programs will depend on selecting highly repellent chemicals, but also on optimizing field deployment strategies. Thymol provides good short-term repellency and is slightly toxic to *D. suzukii* at the rate tested, and Bio-flakes^®^ are easy to spread throughout fields, with flakes landing near ripening low-growing berries. However, the amount of thymol released per hour by Bio-flakes^®^ in this study may not have been sufficient to provide control for more than a few days, and therefore higher densities or larger sizes of Bio-flakes^®^ should be tested. In addition, effects of thymol and other potential repellents should be assessed on beneficial, non-target organisms, similar to testing of geosmin and 1-octen-3-ol [30] Overall, methods and technologies to increase longer-term repellent concentrations, particularly proximal to ripening fruit, as well as testing of push-pull systems tailored to specific crops, regions and fruit ripening periods [25] should be the focus of future investigations.

## Figures and Tables

**Table 1:** Essential oils and their major (>5%) compounds tested for *Drosophila suzukii* repellency.

Essential Oil	Major Compounds (%)			Total %	References
Citronella	citronellal (34)	geraniol (22)	citronellol (12)	68	[32,33]
Eucalyptus	1,8 cineole (68)	α—terpineol (12)		80	[34]
Geranium	citronellol (34)	geraniol (16) ^1^	citronellyl formate (9)	71	[35,36,37]
	isomenthone (7)	linalool (5)			
Peppermint	menthol (45)	menthone (14)		59	[38,39,40]
Thyme	thymol (35)	γ—terpinene (20)	ρ—cymene (20)	75	[41,42]

^1^ Geraniol was only tested individually at 22%, the concentration that it occurs in citronella oil.

**Table 2:** Amounts (mg) of four compounds tested as oviposition deterrents against *Drosophila suzukii* in laboratory choice and no-choice bioassays and number of replicates of each treatment.

Compounds	Treatments		
	Thymol	Citronellol	Four-Compound Blend
Thymol	11.56	0.68	3.5
Citronellol	0.68	11.56	3.4
Menthol	0.68	0.68	4.5
Geraniol	0.68	0.68	2.2
**Total**	13.6	13.6	13.6
Replicates			
*Choice*	10	10	10
*No-choice* 23–24 July ^1^	4	5	5
*No-choice* 30–31 July	6	7	5

^1^ Five and seven control (untreated) replicates on 23–24 July and 30–31 July, respectively.
